# New insights into an X-traordinary viral protein

**DOI:** 10.3389/fmicb.2014.00126

**Published:** 2014-04-08

**Authors:** Torsten Schaller, Hélène Bauby, Stéphane Hué, Michael H. Malim, Caroline Goujon

**Affiliations:** ^1^Department of Infectious Diseases, King's College LondonLondon, UK; ^2^Department of Infection, Division of Infection and Immunity, Centre for Medical Molecular Virology, University College LondonLondon, UK

**Keywords:** vpx, HIV-2, SIVsmm, SAMHD1, myeloid cells, HIV-1, restriction factor, interferon type I

## Abstract

Vpx is a protein encoded by members of the HIV-2/SIVsmm and SIVrcm/SIVmnd-2 lineages of primate lentiviruses, and is packaged into viral particles. Vpx plays a critical role during the early steps of the viral life cycle and has been shown to counteract SAMHD1, a restriction factor in myeloid and resting T cells. However, it is becoming evident that Vpx is a multifunctional protein in that SAMHD1 antagonism is likely not its sole role. This review summarizes the current knowledge on this X-traordinary protein.

Vpx was initially identified as an HIV-2 (human immunodeficiency virus type 2)/SIVsmm (simian immunodeficiency virus infecting sooty mangabey monkey) protein of 12–16 kDa, which is incorporated into viral particles (Franchini et al., 1988; Henderson et al., 1988; Kappes et al., 1988; Yu et al., 1988). In addition to viruses from the HIV-2/SIVsmm lineage of primate lentiviruses, this gene is also found in viruses from the SIVrcm (infecting red-capped mangebey)/SIVmnd-2 (infecting mandrill) lineage (Beer et al., 2001; Hu et al., 2003). It is homologous to the *vpr* gene, found in every lineage of primate lentiviruses (Tristem et al., 1990). Vpx was rapidly shown to be dispensable for viral replication in immortalized lymphocytic cell lines, such as HUT78, CEM, or SupT1 (Yu et al., 1988; Guyader et al., 1989; Hu et al., 1989; Shibata et al., 1990; Gibbs et al., 1994; Park and Sodroski, 1995), and in the monocytic cell lines HL60 and U937 (Guyader et al., 1989; Hu et al., 1989). In contrast, *vpx* deletion led to a strong replication defect in monocyte-derived macrophages (MDMs) (Yu et al., 1991; Gibbs et al., 1994; Park and Sodroski, 1995; Fletcher et al., 1996; Ueno et al., 2003). In addition, *vpx* deletion led to SIVmac (infecting rhesus monkey) and HIV-2 replication defects in activated peripheral blood mononuclear cells (PBMCs) or primary T cells, especially at low viral inputs (Guyader et al., 1989; Kappes et al., 1991; Yu et al., 1991; Akari et al., 1992; Gibbs et al., 1994; Kawamura et al., 1994; Park and Sodroski, 1995; Ueno et al., 2003). Vpx was shown to be important for HIV-2 replication in HSC-F cells, a simian lymphocytic cell line (Ueno et al., 2003). Vpx is packaged into viral particles *via* an interaction with the p6 domain of Gag (Wu et al., 1994; Accola et al., 1999; Selig et al., 1999) and is associated with mature viral cores (Kewalramani and Emerman, 1996). This suggested that Vpx could participate in the early steps of infection. Comparisons of virus associated proteins suggested that Vpx from SIVmac and HIV-2 are packaged in equimolar amounts to Gag (Henderson et al., 1988), although the exact number of molecules packaged per virion has not been determined.

Vpx localizes to the nucleus in transfected cells (Depienne et al., 2000; Mahalingam et al., 2001; Belshan and Ratner, 2003), and this is conferred by a C-terminal non-canonical nuclear localization signal (NLS) (65-SYTKYRYL-72) (Figure 1) (Belshan and Ratner, 2003; Rajendra Kumar et al., 2003), as well as a potential second N-terminal NLS (Singhal et al., 2006a). Whether Vpx shuttles between the cytoplasm and nucleus due to a nuclear export signal remains controversial (Belshan and Ratner, 2003; Singhal et al., 2006b). Likewise Vpx phosphorylation has been proposed to regulate its nuclear import (Rajendra Kumar et al., 2005) but other studies failed to detect this post-translational modification (Franchini et al., 1988; Belshan et al., 2006). By virtue of its karyophilic properties, Vpx was proposed to play a critical role in the nuclear import of viral reverse transcription complexes in non-dividing cells, such as MDMs and arrested U937 cells (Pancio et al., 2000; Mahalingam et al., 2001; Rajendra Kumar et al., 2003). Indeed the replication defect of viruses lacking Vpx (or bearing non-karyophilic mutated versions of Vpx) correlated with the absence of 2-LTR circles (a surrogate marker for viral DNA nuclear entry) (Fletcher et al., 1996; Pancio et al., 2000; Ueno et al., 2003; Belshan et al., 2006).

**Figure 1:**
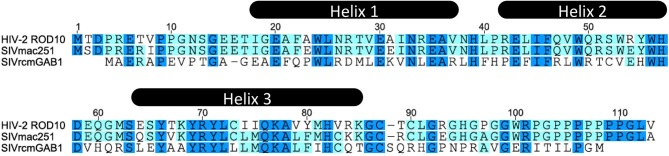
**HIV-2 ROD, SIVmac, and SIVrcm Vpx aminoacid sequence alignment**.

Later studies using lentiviral vectors and single-round infections confirmed a cell-type dependent effect of Vpx and a role in the early events of infection. Vpx is essential for transduction of monocyte-derived dendritic cells (MDDCs) with SIVmac based-lentiviral vectors (Mangeot et al., 2002). Surprisingly, when brought *in trans via* virus-like particles (VLPs), Vpx increases HIV-1 transduction of MDDCs and MDMs but not activated T cells (Goujon et al., 2006). This positive effect of Vpx in MDDCs was directly correlated with an increase in viral DNA accumulation, which was observed not only with SIVmac but also with heterologous retroviral vectors, derived from HIV-1, feline immunodeficiency virus (FIV) and murine leukemia virus (MLV) (Goujon et al., 2007). Of note, in the case of MLV, Vpx rescued viral DNA accumulation but not 2-LTR circle formation (Goujon et al., 2007; Gramberg et al., 2013), consistent with a nuclear-entry block to MLV infection in non-dividing cells (Roe et al., 1993; Lewis and Emerman, 1994). Vpx was later shown to favor HIV-2/SIVsmm DNA accumulation in MDMs (Fujita et al., 2008; Srivastava et al., 2008; Bergamaschi et al., 2009).

Animal studies showed that Vpx is crucial for SIVsmm PBj and SIVmne (infecting pig-tailed macaque) replication and spread in a pig-tailed macaque model. SIVsmm PBj *vpx* mutant virus replicated to a considerably lower extent and showed much reduced kinetics as compared to wild-type virus (Hirsch et al., 1998). In addition, the mutant virus was outcompeted when inoculated together with wild-type SIVsmm PBj (Hirsch et al., 1998). *In vitro*, SIVmne *vpx* mutants infected pig-tailed macaque PBMCs to comparable levels to that of wild-type virus, but showed significantly reduced infectivity in MDMs (Belshan et al., 2012). Another study reported a less prominent effect of Vpx in rhesus monkeys infected with SIVmac239, with delayed viral kinetics for *vpx* deficient virus but AIDS development nonetheless (Gibbs et al., 1995).

The fact that Vpx could act *in trans* on other retroviruses strongly suggested at the time that Vpx modulates the cellular environment to increase cell permissivity to infection, possibly by preventing the action of an inhibitory factor. The first evidence of the existence of such a dominant inhibitory factor in myeloid cells came from the use of heterokaryons generated between permissive (COS) cells and restrictive cells (MDMs) (Sharova et al., 2008). Unlike COS cells, both MDMs and COS-MDMs heterokaryons restricted SIVsmm PBj in the absence of Vpx at the level of viral DNA accumulation. The same authors also observed a dominant restriction phenotype in heterokaryons formed between primary monocytes and HeLa cells (Kaushik et al., 2009).

## Hijacking the DCAF1/DDB1/CUL4A E3 ubiquitin ligase

In parallel with the aforementioned viral and cell biology experiments, proteomic studies yielded some fundamental insights. Initially, Le Rouzic et al. discovered that HIV-1 Vpr recruits the damage-specific DNA binding protein 1 (DDB1)-Cullin 4A (CUL4A) E3 ubiquitin ligase complex through the binding of a known interactor of Vpr, VprBP (Zhao et al., 1994; Le Rouzic et al., 2007). VprBP had previously been identified in a screen as the substrate recruiting module of DDB1-CUL4A-RBX1/ROC1 complexes, and renamed DDB1-CUL4A-associated factor 1 (DCAF1) (Angers et al., 2006). Using yeast two-hybrid, Le Rouzic et al. also showed that Vpx from SIVmac, similarly to HIV-1 Vpr, was able to bind VprBP/DCAF1 (Le Rouzic et al., 2007). This interaction was soon confirmed in mammalian cells (Goujon et al., 2008; Sharova et al., 2008; Srivastava et al., 2008; Bergamaschi et al., 2009). RNAi-mediated depletion of DCAF1 or DDB1 profoundly reduced SIVmac as well as HIV-2 infection of macrophages (Sharova et al., 2008; Srivastava et al., 2008; Bergamaschi et al., 2009). This invoked a model by which Vpx was hijacking a DCAF1-DDB1-CUL4A E3 ubiquitin ligase to induce the degradation of a myeloid cell specific restriction factor that prevents viral DNA accumulation (Sharova et al., 2008; Srivastava et al., 2008; Bergamaschi et al., 2009).

## SAMHD1 antagonism

To isolate the cellular factor(s) limiting HIV-1 infection in myeloid cells, the groups of Monsef Benkirane and Jacek Skowronski exploited similar proteomic approaches, using tandem affinity purification combined with mass spectrometry to identify Vpx binding partners (Hrecka et al., 2011; Laguette et al., 2011). Both labs identified sterile alpha motif (SAM) and HD-domain-containing protein 1 (SAMHD1) as one of the major binding partners of SIVmac Vpx. SAMHD1 silencing phenocopied the effect of Vpx-containing VLPs in that it rescued HIV-1 infection of both differentiated THP-1 monocytic cells and MDDCs (Laguette et al., 2011). Both studies showed that HIV-2/SIVmac Vpx induced SAMHD1 proteasomal degradation (Hrecka et al., 2011; Laguette et al., 2011). Importantly, SAMHD1 antagonism is a function conserved in all clades of Vpx proteins, in a species specific way (Lim et al., 2012). In certain SIVs devoid of a Vpx protein, Vpr can antagonize SAMHD1. For instance, Vpr from SIVdebCM5 (infecting De Brazza's monkeys), SIVagm (African green monkeys), and SIVmus1 (mustached monkeys) can degrade SAMHD1 of their natural host when expressed in human cells, whereas Vpr from HIV-1 and SIVcpz (chimpanzee) cannot (Lim et al., 2012).

SAMHD1 is a 626 amino acid protein that consists of an amino-terminal SAM domain, a central HD domain and a C-terminal uncharacterized domain (Li et al., 2000; Liao et al., 2008). SAMHD1 is a deoxynucleoside-triphosphate (dNTP) phosphohydrolase (Goldstone et al., 2011; Powell et al., 2011; Yan et al., 2013), which reduces the pool of dNTPs available for reverse transcription both in myeloid cells (Lahouassa et al., 2012; St Gelais et al., 2012) and in resting T cells (Bailes et al., 2012; Descours et al., 2012). These observations therefore explain why the provision of exogenous deoxyribonucleosides (dN) to resting T cells, which have long been known to restrict lentiviral infection at several steps of the life cycle (Pan et al., 2013), increases the accumulation of viral DNA (Korin and Zack, 1999).

Crosslinking experiments show that SAMHD1 forms oligomers in cells and it has been proposed that the enzymatically active form of SAMHD1 is a tetramer and that tetramerization is driven by dGTP binding to the allosteric sites (Ji et al., 2013; Yan et al., 2013). Knock-out mice analysis have recently confirmed that SAMHD1 functions as a dNTPase *in vivo* as these mice show elevated levels of intracellular dNTPs in DCs isolated from bone marrow (Behrendt et al., 2013; Rehwinkel et al., 2013). Therefore, SAMHD1 reduces cellular dNTP levels and this impacts reverse transcription most significantly in situations where dNTP levels are naturally lower, such as post-mitotic or non-dividing cells.

Mutations in SAMHD1 are associated with the genetic neurodegenerative disorder Aicardi-Goutières syndrome (AGS), which is characterized by the excessive production of type 1 interferon (IFN) in the cerebrospinal fluid and resembles congenital infection (Rice et al., 2009; Chahwan and Chahwan, 2012). Interestingly, CD14+ cells from AGS patients with mutations in *SAMHD1* are more susceptible to HIV-1 infection than cells from healthy controls (Berger et al., 2011a). This shows the importance of SAMHD1 for preventing HIV-1 infection in monocytes, well known to be naturally refractory to HIV-1 infection *in vitro* (Sonza et al., 1996; Neil et al., 2001; Triques and Stevenson, 2004).

In addition to a variety of retroviruses (Gramberg et al., 2013; Sze et al., 2013), SAMHD1 blocks replication of DNA viruses, such as vaccinia virus and herpex simplex virus 1 (Hollenbaugh et al., 2013; Kim et al., 2013). SAMHD1 is also active against retroelements (Zhao et al., 2013), suggesting that increased retrotransposition might be leading to immune sensing of DNA and activation of signaling pathways in AGS patients with *SAMHD1* mutations, as proposed for the 3'-5' exonuclease TREX1 (three prime repair exonuclease) (Stetson et al., 2008; Beck-Engeser et al., 2011). Indeed, mutations in *TREX1* can cause AGS (Crow et al., 2006) and in *Trex1*-deficient mice, reverse transcribed DNA from endogenous retroelements accumulated and stimulated intrinsic immune responses (Stetson et al., 2008). Interestingly, Zhao et al. showed that both endogenous and overexpressed SAMHD1 prevented LINE-1 (long interspersed element-1) retrotransposition in 293T cells (Zhao et al., 2013). This effect of SAMHD1 on retrotransposition was not dependent on its catalytic activity and was counteracted by Vpx. Therefore, infection by lentiviruses encoding a SAMHD1 antagonist (either Vpx or Vpr) might lead to increased replication of endogenous retroelements and a possible impact on host genome stability.

SAMHD1 is expressed at similar levels in MDMs, resting CD4+ T cells and in activated CD4+ T cells (Bailes et al., 2012; Descours et al., 2012), but does not block HIV-1 infection in the latter. The discrepancy of SAMHD1's antiviral function in cycling vs. non-cycling cells led several groups to investigate cell cycle dependent determinants for both the antiviral and dNTPase activities of SAMHD1. SAMHD1 was found to interact with and be phosphorylated by the cell cycle regulator cyclin-dependent kinase 1 (CDK1) in proliferating cells, and phosphorylation at the residue T592 has been shown to prevent lentiviral restriction (Cribier et al., 2013; White et al., 2013). CDK1 is inactive in resting cells, suggesting that the cell cycle progression correlates with SAMHD1's antiviral activity. In line with this, a phosphorylation-defective mutant of SAMHD1 was antiviral both in resting and in dividing U937 cells (Cribier et al., 2013). In addition, the phosphomimetic SAMHD1 mutant T592E was unable to restrict HIV-1 infection (Welbourn et al., 2013; White et al., 2013). However, phosphorylation did not affect the ability of SAMHD1 to hydrolyse dNTPs in an *in vitro* dNTPase assay (Welbourn et al., 2013) or in differentiated U937 monocytic cells (White et al., 2013). Although phosphorylated and lacking antiviral activity in cycling cells, SAMHD1's role in dNTP metabolism in these cells remains unclear. Cycling cells contain high levels of dNTPs (Diamond et al., 2004), therefore *de novo* dNTP synthesis likely compensates for any potential effect of SAMHD1. The fact that SAMHD1 mutant T592E lacks antiviral activity while being an active dNTPase suggests the existence of a potential dNTPase-independent restriction mechanism. Supporting the notion that SAMHD1's influence on infection may be more complex, it has been suggested that SAMHD1 interacts with nucleic acids, specifically ssRNA and ssDNA (Goncalves et al., 2012; Tungler et al., 2013) and that it possesses a nuclease activity (Beloglazova et al., 2013).

SAMHD1 is localized in the nucleus of differentiated cells. However, disruption of its NLS does not affect antiviral activity (Rice et al., 2009; Brandariz-Nunez et al., 2012; Hofmann et al., 2012), yet results in a relative resistance to SIVmac Vpx-mediated degradation (Brandariz-Nunez et al., 2012; Hofmann et al., 2012; Wei et al., 2012; Guo et al., 2013). Given that Vpx efficiently interacts with both nuclear and cytoplasmic SAMHD1 (Hofmann et al., 2012), it is difficult to understand why cytoplasmic SAMHD1 is less sensitive to Vpx-induced degradation. It is possible for example that factors of the DCAF1-DDB1-CUL4A E3 ubiquitin ligase machinery are limiting in the cytoplasm, or that differences in SAMHD1's post-translational modifications prevent cytoplasmic SAMHD1 degradation. Alternatively there might be differences in ubiquitination or deubiquitination processes between nuclear and cytoplasmic SAMHD1.

Intriguingly, in contrast to what is observed in MDDCs, wild-type SAMHD1 from myeloid and plasmacytoid DCs (mDCs and pDCs, respectively), was shown to be resistant to Vpx-induced degradation (Bloch et al., 2014). The sensitivity to Vpx-induced degradation of SAMHD1 as well as the Vpx effect on HIV-1 infectivity could be partially restored by blocking IFN signaling using neutralizing antibodies (Bloch et al., 2014). This suggests that IFN-induced factors might prevent SAMHD1 degradation in mDCs and pDCs or that SAMHD1 localization might be modified following IFN exposure, rendering it resistant to Vpx-mediated degradation. In line with this, Dragin et al. observed a reduced sensitivity of SAMHD1 to Vpx-mediated degradation in IFN-treated THP-1, suggesting that IFN-stimulated genes may participate in this process (Dragin et al., 2013). In addition, IFN treatment did not modify SAMHD1 localization (Dragin et al., 2013). In contrast, we have observed an efficient degradation of SAMHD1 in IFN-treated THP-1 cells (Goujon et al., 2013), but the reasons for these differences are currently unknown. Type 1 IFN treatment reduced SAMHD1 phosphorylation levels of residue T592 in MDMs and MDDCs, suggesting the existence of IFN-inducible phosphatase(s) activating SAMHD1 (Cribier et al., 2013). The identification of this/these phosphatase(s) would improve our understanding of the regulation of SAMHD1's antiviral activity.

## Structural insights

**Figure 2:**
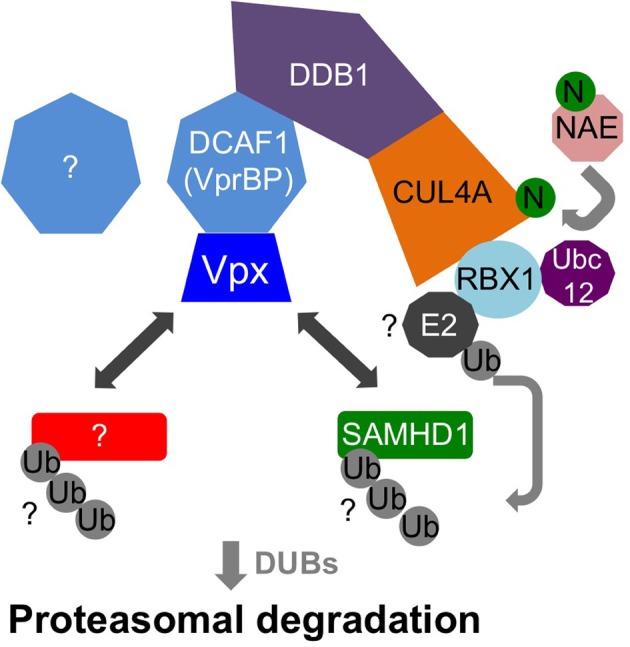
**Model of DCAF1-DDB1-CUL4A recruitment by Vpx**. Vpx hijacks the DDB1-CUL4A-RBX1 E3 ubiquitin ligase complex through recruitment of DCAF1 (also known as VprBP). Vpx targets (i.e., SAMHD1 and possibly unknown proteins) are ubiquitinated through the activity of RBX1 and an E2 ligase that interacts with RBX1. In addition, RBX1 interacts with the ubiquitin-conjugating enzyme UBC12, which is important for neddylation of CUL4A through transfer of the NEDD8 group from NEDD8 activating enzyme (NAE).

**Figure 3:**
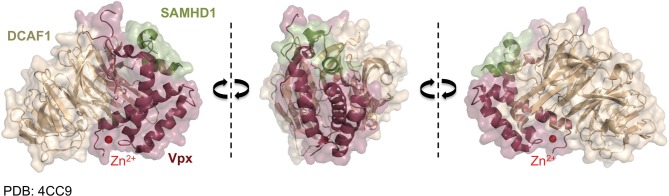
**Vpx/SAMHD1 CtD/DCAF1 CtD complex structure**. Shown is the co-crystal structure of DCAF1 CtD (brown) with SAMHD1 CtD (green) in complex with Vpx from SIVsmm (dark red) (Schwefel et al., 2014) (PDB:4CC9) from three different sites. The zinc ion coordinated by Vpx is shown in red.

**Figure 4:**
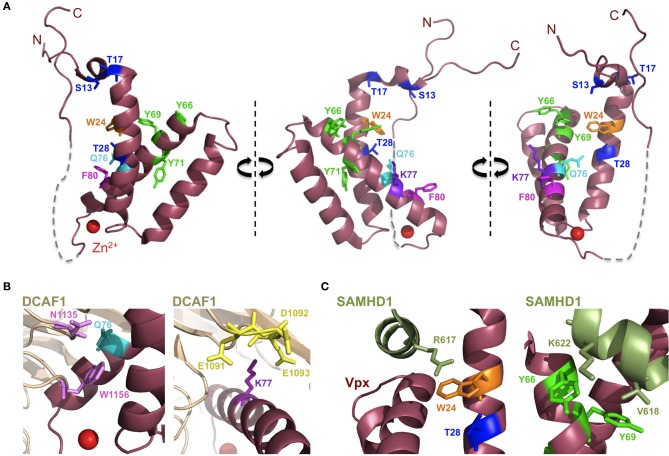
**Vpx structure. (A)** Depicted is the structure of Vpx from SIVsmm as obtained in the co-crystallization with the C-terminus of DCAF1 and the C-terminus of SAMHD1 (both not shown) with critical amino acids highlighted (PDB:4CC9) (Schwefel et al., 2014). SIVsmm Vpx comprises an anti-parallel bundle of three helices, resembling the shape of a V. Vpx coordinates a zinc ion (red) with amino acids H39, H82, C87, and C89. The structure between Vpx residues 90–100 is absent (dashed gray line). **(B)** Critical aminoacid residues in the interface between DCAF1 and Vpx are highlighted. Vpx Q76 residue (cyan) may form hydrogen bonds with N1135 and W1156 residues (lilac) of DCAF1. The acidic region in DCAF1 comprising E1091, D1092 and E1093 residues (yellow) may interact with positively charged K77 residue (purple) of Vpx. **(C)** Critical amino acid residues for the interaction of SAMHD1 and Vpx are highlighted. SAMHD1 R617 residue (light green) stacks with the indole ring of Vpx W24 residue (orange). Vpx Y66 and Y69 residues (fluorescent green) are in close proximity to SAMHD1 K622 and V618 residues (light green), the latter being positively selected (Laguette et al., 2012).

Vpx recruits the DCAF1-DDB1-CUL4A E3 ubiquitin ligase complex to induce SAMHD1's proteasomal degradation *via* an interaction with its C-terminal domain (Ahn et al., 2012) (Figure 2). The crystal structure of a complex between SIVsmm Vpx, the C-terminal domain of the ubiquitin-ligase adaptor DCAF1 (DCAF1-CtD) and the Vpx-binding, C-terminal domain of SAMHD1 (SAMHD1-CtD, residues 582-626) was revealed recently (Figure 3) (Schwefel et al., 2014). This structure shows that Vpx is composed of a three-helix bundle stabilized by a zinc finger motif (formed of residues H39, H82, C87 and C89) (Figure 4A) and extensively interacts with DCAF1-CtD (Figure 4B), the latter forming a seven-bladed β -propeller disc-shaped molecule. Both proteins offer a shared interface used to bind SAMHD1-CtD. A model of the hijacked DCAF1-DDB1-CUL4A complex in association with the RBX1 RING module (RING-box protein 1), generated using previously determined structures, clearly showed that SAMHD1 is positioned in the vicinity of the RBX1 RING module, allowing accessibility to ubiquitination (Schwefel et al., 2014) (Figure 2). The SAMHD1 target sites for Vpx-induced ubiquitination have not been determined yet. In addition to ubiquitination, neddylation is required for SAMHD1 degradation, consistent with the importance of NEDD8 transfer to CUL4A through UBC12 (Hofmann et al., 2013). It would be of interest to understand the structural consequences of Vpx interaction with tetrameric SAMHD1. There is some evidence from surface plasmon resonance experiments that Vpx interaction with SAMHD1 causes the disassembly of enzymatically active SAMHD1 oligomers and interferes with SAMHD1 enzymatic activity prior to its degradation (Delucia et al., 2013). This suggests a model where proteasomal degradation may be the consequence and not the initiating event in Vpx-mediated SAMHD1 antagonism.

Extensive mutagenesis of Vpx has shed light into the critical residues of Vpx (Table 1 and Figure 4). Notably, N-terminal Vpx amino acids are essential to rescue virus infectivity in myeloid cells and SAMHD1 degradation (Goujon et al., 2008; Gramberg et al., 2010; Ahn et al., 2012; Fregoso et al., 2013). Of major interest, the phenotypes of some well characterized Vpx mutants are explained by the structure (Schwefel et al., 2014). For instance, the Vpx Q76A mutant is unable to bind DCAF1 and degrade SAMHD1 (Srivastava et al., 2008; Bergamaschi et al., 2009; Hrecka et al., 2011). The structure shows that this defect may be due to disrupted hydrogen bonds between Q76 and residues N1135 and W1156 of DCAF1 (Figure 4B) (Schwefel et al., 2014). Similarly, residue K77, critical for DCAF1 binding and Vpx activity (Bergamaschi et al., 2009), is integral to an extensive salt-bridge network that links residues E1091-D1092-E1093 of DCAF1 with residues R70, Y69 and Y66 in helix 3 of Vpx (Figure 4B). In addition, Vpx W24A mutant was shown to have lost SAMHD1 antagonism while being able to bind to DCAF1 (Wei et al., 2012). This phenotype is explained by a stacking between residues W24 of Vpx and R617 of SAMHD1 (Figure 4C) (Schwefel et al., 2014). In line with this, SAMHD1 R617 mutants completely lost sensitivity to Vpx-mediated degradation while maintaining antiviral activity (Schwefel et al., 2014).

**Table 1:** **Phenotype of Vpx mutants**.

**Mutant**	**Virus**	**Phenotype**	**References**
N12A	SIVmac	No infection rescue of HIV-1 in MDMs	Gramberg et al., 2010; Ahn et al., 2012
		Partial SAMHD1 degradation	
S13A	SIVmac	Rescue SIVmac and HIV-1 in MDMs and MDDCs	Goujon et al., 2008; Gramberg et al., 2010; Berger et al., 2012
		SAMHD1 degradation	
E15A	SIVmac	No rescue of HIV-1 in MDMs	Gramberg et al., 2010; Ahn et al., 2012
		Partial SAMHD1 degradation	
E16A	SIVmac	No rescue of HIV-1 in MDMs	Gramberg et al., 2010; Ahn et al., 2012
		Partial SAMHD1 degradation	
T17A	SIVmac	No rescue of SIVmac in MDDCs but partial activity on HIV-1, no rescue in MDMs	Goujon et al., 2008; Gramberg et al., 2010; Ahn et al., 2012
		Partial SAMHD1 degradation	
E20A	SIVmac	Rescue of HIV-1 in MDMs	Gramberg et al., 2010
W24A	SIVmac	No infection rescue, interaction with DCAF1	Wei et al., 2012
N26A	SIVmac	Rescue of SIVmac and HIV-1 in MDDCs	Goujon et al., 2008
R27A	SIVmac	Rescue of HIV-1 in MDMs	Gramberg et al., 2010
T28A	SIVmac	No rescue of SIVmac in MDDCs but partial activity on HIV-1	Goujon et al., 2008
V29S	SIVmac	Reduced interaction with DCAF1	Wei et al., 2012
		Interaction with SAMHD1 but no degradation, no infection rescue	
STT13,17,28A	SIVmac	No infection rescue in MDDCs with SIVmac or HIV-1, no SAMHD1 degradation	Goujon et al., 2008
I32S	SIVmac	No infection rescue, no interaction with DCAF1, no degradation of SAMHD1	Wei et al., 2012
H39A	SIVmac, HIV-2 GH-1	No infection rescue, no interaction with DCAF1, no degradation of SAMHD1	Goujon et al., 2008; Wei et al., 2012
W49,53,56A	SIVmac	No infection rescue in MDDCs with SIVmac or HIV-1	Goujon et al., 2008; Berger et al., 2012
		No degradation of SAMHD1	
S52A	SIVmac HIV-2 GH-1	Infection rescue in MDDCs with SIV or HIV-1, HIV-2GL-AN replication in MDDCs	Goujon et al., 2008; Berger et al., 2012
		SAMHD1 degradation	
S63,65A	SIVmac	Infection rescue in MDDCs with SIV or HIV-1	Goujon et al., 2008
KK84,85A	SIVmac, HIV-2	Infection rescue in MDDCs with HIV-1, SAMHD1 degradation	Goujon et al., 2008; Berger et al., 2012
	GH-1	Slightly reduced activity for SIVmac and HIV-2GL-AN replication in MDDCs	
KK84,85R	SIVsm PBj1.9	Defect in DNA accumulation in MDMs	Sharova et al., 2008
K77A	SIVsmPBj	No infection rescue, no interaction with DCAF1	Bergamaschi et al., 2009
KK68,77A or R	SIVmac SIVsm PBj1.9	No infection rescue in MDDCs or MDMs infection with SIVmac, SIVSM PBj1.9 or HIV-1	Goujon et al., 2008; Sharova et al., 2008; Berger et al., 2012
		No degradation of SAMHD1	
KKKR68,77,84,85A or R	HIV-2 GH-1, SIVsm PBj1.9	No infection of MDMs, no replication of HIV-2 GL-AN in MDDCs	Goujon et al., 2008; Sharova et al., 2008
Y66,69,71A	SIVmac, SIVmne	No infection rescue, no interaction with DCAF1, no degradation of SAMHD1	Goujon et al., 2008; Belshan et al., 2012; Berger et al., 2012
Q76A or R	SIVmac, HIV-2 ROD, HIV-2	No infection rescue, no interaction with DCAF1, no degradation of SAMHD1	Srivastava et al., 2008; Hrecka et al., 2011; Berger et al., 2012
	GH-1	BUT rescue of HIV-1 from IFN block	Bergamaschi et al., 2009; Laguette et al., 2011; Pertel et al., 2011 Wei et al., 2012
F80A	SIVmac	No infection rescue, no interaction with DCAF1, no degradation of SAMHD1	Srivastava et al., 2008; Laguette et al., 2011; Pertel et al., 2011
		BUT rescue of HIV-1 from IFN block	
GC86,87A	SIVmac	No rescue of MDDCs infection with SIVmac but partial activity on HIV-1	Goujon et al., 2008
ΔPro	SIVmac, HIV-2 GH-1, HIV-2	Rescue of HIV-1 infection, slight activity loss on SIVmac, SAMHD1 degradation	Pancio et al., 2000; Goujon et al., 2008; Berger et al., 2012
	ROD	No replication of HIV-2GL-AN or HIV-2rod in MDDCs and MDMs	

*Non-exhaustive list of characterized Vpx mutants, summarizing their activity either in single-cycle infection with SIVmac and HIV-1 or in replication (in MDMs and/or MDDCs), and their ability to interact with DCAF1 and SAMHD1, and to degrade the latter*.

## Additional roles of Vpx?

The fact that *vpx* deletion leads to some replication defects in activated PBMCs or primary T cells (Guyader et al., 1989; Kappes et al., 1991; Yu et al., 1991; Akari et al., 1992; Gibbs et al., 1994; Kawamura et al., 1994; Park and Sodroski, 1995; Ueno et al., 2003) strongly suggests that Vpx might have other functions beyond counteracting SAMHD1, which is only relevant in myeloid and resting T cells. As mentioned above, it has been proposed that Vpx participates in the nuclear import of viral reverse transcription complexes (Fletcher et al., 1996; Pancio et al., 2000; Ueno et al., 2003; Belshan et al., 2006). Possibly in favor of such an additional role of Vpx, some Vpx mutants have a more profound effect on MDDCs transduction with SIVmac lentiviral vectors than with HIV-1 (e.g., T17A, T28A, or GC86,87A Vpx mutants which fail to rescue SIVmac infection but improve HIV-1 infection by more than one order of magnitude Goujon et al., 2008; Table 1). Moreover, a Vpx mutant devoid of the C-terminus proline-rich region is still able to efficiently degrade SAMHD1 and to rescue HIV-1 in single-cycle infection of MDDCs (Goujon et al., 2008; Berger et al., 2012), but does not support HIV-2 replication in MDMs or MDDCs (Pancio et al., 2000; Goujon et al., 2008). Finally, Vpx was proposed to increase HIV-1 infectivity in a DCAF1-independent way both in IFN-treated MDDCs and in THP-1 cells, suggesting additional function(s) beyond SAMHD1 degradation (Goujon et al., 2008; Pertel et al., 2011; Reinhard et al., 2014).

### APOBEC3A antagonism?

In addition to the well-documented interaction with SAMHD1, Vpx interacts with a member of the apolipoprotein B mRNA-editing enzyme catalytic polypeptide-like 3 (APOBEC3) family of cytidine deaminases, namely APOBEC3A. APOBEC3 family members, exemplified by APOBEC3G, are potent restriction factors (reviewed in Malim and Bieniasz, 2012). HIV-1 prevents APOBEC3G/F/D/H action through proteasomal degradation induced by the Vif accessory protein. APOBEC3A affects various viruses and retroelements, such as human papilloma virus (Vartanian et al., 2008), adeno-associated virus (AAV) (Chen et al., 2006), and retrotransposons (Bogerd et al., 2006; Chen et al., 2006; Muckenfuss et al., 2006). Interestingly, APOBEC3A expression levels have been linked to myeloid cell restriction of HIV-1 infection (Peng et al., 2007). In line with this, APOBEC3A silencing increased the ability of HIV-1 to replicate in MDMs (Peng et al., 2007; Berger et al., 2011b). Two independent groups have shown that HIV-2/SIVsmm Vpx interacts with human APOBEC3A using co-immunoprecipitation of overexpressed proteins (Berger et al., 2010, 2011b). Both groups reported a decreased stability of APOBEC3A in the presence of Vpx. Similarly, wild-type SIVsmm replication led to slightly decreased APOBEC3A expression levels in monocytes compared to Vpx-deficient viruses (Berger et al., 2010). These data led to the hypothesis that Vpx might enhance infection of myeloid cells by inducing APOBEC3A degradation. However, the precise contribution of APOBEC3A to the resistance of HIV-1 infection in myeloid cells is difficult to evaluate, as SAMHD1 imposes a strong barrier to infection in these cells and is counteracted by Vpx. It would be crucial to separate, if possible, the two activities of Vpx, for instance by identifying mutants of Vpx retaining the ability of interacting with APOBEC3A but not SAMHD1 (or vice versa), and evaluating their effect in myeloid cells.

### Antagonism of interferon-induced factor(s)?

It has been known for decades that type 1 IFN treatment potently decreases HIV-1 infection in some immortalized cell lines and in primary cells, such as MDMs and MDDCs (Kornbluth et al., 1989; Shirazi and Pitha, 1992; Baca-Regen et al., 1994; Cheney and McKnight, 2010; Goujon and Malim, 2010). Interestingly, this IFN-induced block is primarily exerted at the level of viral DNA accumulation (Shirazi and Pitha, 1993; Baca-Regen et al., 1994; Cheney and McKnight, 2010; Goujon and Malim, 2010), which is exactly the step at which SAMHD1 acts (Hrecka et al., 2011; Laguette et al., 2011). Strikingly, Vpx is still capable of enhancing HIV-1 infection in IFN-treated myeloid cells and the magnitude of the infectivity enhancement is increased in this context (Gramberg et al., 2010; Pertel et al., 2011; Goujon et al., 2013). SAMHD1 was reported to be IFN-inducible in some cell types, such as primary monocytes (Berger et al., 2011a). This suggested that SAMHD1 might be playing a role in the IFN block to HIV-1 infection. But neither SAMHD1 expression nor dNTP intracellular concentrations seem to be regulated by IFN treatment in MDMs or MDDCs (St Gelais et al., 2012; Dragin et al., 2013; Goujon et al., 2013). Furthermore, RNAi-mediated SAMHD1 silencing fails to rescue myeloid cell infection in the presence of IFN (Dragin et al., 2013; Goujon et al., 2013). In addition, Q76A Vpx mutant was still able to substantially rescue IFN-treated MDDC infection (Pertel et al., 2011; Reinhard et al., 2014), although it was unable to bind DCAF1 (Pertel et al., 2011) and induce SAMHD1 degradation (Hrecka et al., 2011). Whereas exogenous dN and SIVmac Vpx increased the accumulation of HIV-1 late reverse transcription products to the same extent in IFN-treated myeloid cells, Vpx had a higher impact on 2-LTR circle and proviral DNA formation (Reinhard et al., 2014). This feature was not observed with SIVmus and SIVdeb Vpr, despite their ability to degrade human SAMHD1 (Reinhard et al., 2014). Taken together, these data strongly suggest that HIV-2/SIVmac Vpx may be counteracting other myeloid specific, IFN-inducible anti-HIV-1 factor(s). It could be tempting to speculate that APOBEC3A may be one of these. Indeed, APOBEC3A is highly induced by IFN treatment in myeloid cells (Peng et al., 2006; Koning et al., 2009; Refsland et al., 2010) and Vpx is known to interact with APOBEC3A (Berger et al., 2010, 2011b). However, APOBEC3A was not degraded in the presence of Vpx-VLPs following IFN treatment, contrary to SAMHD1, which is potently and rapidly degraded (Dragin et al., 2013). It remains possible that APOBEC3A is sequestered by Vpx rather than degraded in these conditions. Single-genome sequencing of viral DNA following HIV-1 infection of IFN-treated MDMs showed only infrequent editing and no sign of hypermutation, arguing against a major role for APOBEC3 proteins in the IFN block (Koning et al., 2011), though this does not exclude a deamination-independent mechanism. Indeed, APOBEC3A is known to inhibit AAV *via* a deamination-independent mechanism (Narvaiza et al., 2009). Further work will be required to address the exact role of APOBEC3A in the IFN-induced block to HIV-1 infection, and to determine whether Vpx is counteracting additional IFN-induced anti-HIV-1 factors in myeloid cells. Of note, SIVmac lentiviral vectors are still sensitive to the antiviral action of IFN in human primary cells despite the presence of Vpx (Goujon and Malim, 2010; Reinhard et al., 2014). This may reflect the existence of species-specific IFN-induced genes to which SIVmac is sensitive in human cells contrary to HIV-1, and which are not counteracted by Vpx (Bitzegeio et al., 2013; Cordeil et al., 2013).

## Origin and evolution of *vpx*

The HIV-2/SIVsmm and SIVrcm/SIVmnd-2 lineages possess 2 homologous genes to HIV-1/SIVcpz *vpr*: *vpr* and *vpx*. The *vpr* and *vpx* genes are paralogs and are the result of complex duplication and/or recombination of their precursor throughout the diversification of primate lentiviruses. *vpr* has most likely diverged through cross-species transmission, rather than co-evolution with their hosts following ancient infection. To illustrate this, Wertheim and Worobey studied mitochondrial sequences of several species of the African green monkey lineage and their SIVagm and have rejected the hypothesis of co-divergence between SIVagm and its host. Their data support the hypothesis of a host-switching model following a geographical pattern of transmission (Wertheim and Worobey, 2007). Whilst *vpr* is found in all lentiviral groups, *vpx* is found only in two lineages: HIV-2/SIVsmm/SIVmac and SIVrcm/SIVmnd-2 (Beer et al., 2001; Hu et al., 2003). These viruses infect sooty mangabeys, macaques, mandrills and red-capped mangabeys, all of which belong to the same primate family.

The *vpx* genes from the SIVrcm/SIVmnd-2 lineage are quite divergent from other *vpx* genes (only about 30–40% identity at the amino acid level with Vpx from HIV-2/SIVsmm). Nonetheless, the monophyletic nature of the *vpx* clade supports the hypothesis of a single event leading to the birth of *vpx*, rather than multiple events (Hu et al., 2003).

**Figure 5:**
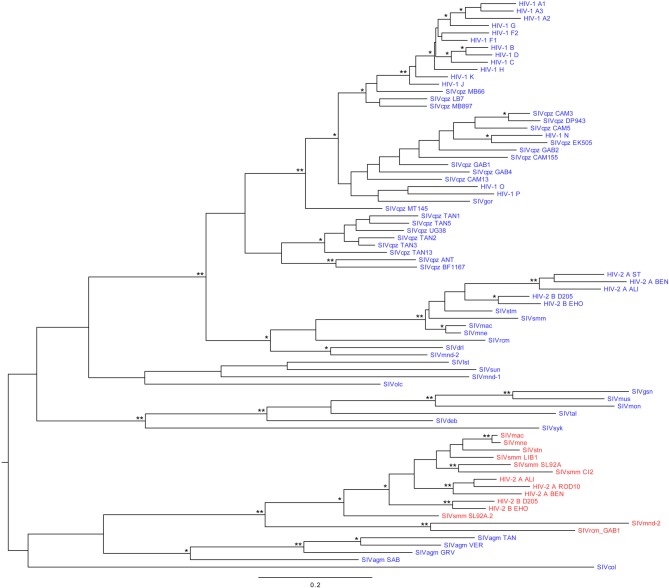
**Maximum likelihood phylogeny of primate lentiviruses *vpr* and *vpx* genes**. *vpr* are represented in blue and *vpx* in red. Branch lengths represent nucleotide substitutions per site, as indicated in the scale. Branch supports are indicated by one asterisk (≥90% confidence) or two asterisks (≥99%). The tree was rooted using the midpoint rooting method. Abbreviations: HIV, human immunodeficiency virus; SIV, simian immunodeficiency virus; cpz, chimpanzee (*Pan troglodytes*); gor, gorilla (*Gorilla gorilla*); stm, stump-tailed macaque (*Macaca arctoides*); smm, sooty mangabey monkey (*Cercocebus atys*); mac, rhesus macaque (*Macaca mulatta*); mne, pig-tailed macaque (*Macaca nemestrina*); rcm, red-capped mangabey (*Cercocebus torquatus*); drl, drill monkey (*Mandrillus leucophaeus*); mnd, mandrill (*Mandrillus sphinx*); lst, L'Hoest's monkey (*Cercopithecus lhoesti*); sun, sun-tailed monkey (*Cercopithecus solatus*); olc, olive Colobus (*Procolobus verus*); gsn, greater spot-nosed monkey (*Cercopithecus nictitans*); mus, mustached monkey (*Cercopithecus cephus*); mon, mona monkey (*Cercopithecus mona*); tal, northern talapoins (*Miopithecus ogouensis*); deb, De Brazza's monkey (*Cercopithecus neglectus*); syk, Sykes' monkey (*Cercopithecus mitis*); agm TAN, tantalus African green monkey (*Chlorocebus tantalus*); agm VER, vervet African green monkey (*Chlorocebus pygerythrus*); agm GRV, grivet African green monkey (*Chlorocebus aethiops*); agm SAB, sabaeus African green monkey (*Chlorocebus aethiops sabaeus*); col, guereza colobus monkey (*Colobus guereza*).

Two hypotheses have been proposed so far to explain the birth of *vpx*. It has first been suggested that *vpx* arose by duplication of the *vpr* ancestor (Tristem et al., 1990). This was supported by sequence similarity and by the fact that both genes are always adjacent to each other in the genome. Alternatively, because *vpx* genes are closely related to *vpr* from SIVagm.Sab (infecting African green monkeys) (Figure 5), it has been proposed by another group that a recombination event between SIVagm and SIVsmm could have led to the acquisition of SIVagm.Sab *vpr* by the latter (Sharp et al., 1996; Hu et al., 2003). In agreement with this, recombination events between those two viruses have been shown before (Jin et al., 1994) and the habitats of African green monkeys and sooty mangabeys are partly overlapping. As the Vpr proteins of the HIV-2/SIVsmm/SIVmac and SIVrcm/SIVmnd-2 lineages are inactive against SAMHD1 (Lim et al., 2012) and as this function was acquired by the other *vpr* genes before the birth of *vpx*, it seems likely that the former Vpr proteins were never able to antagonize SAMHD1. Therefore, the acquisition of *vpr* from SIVagm would have been beneficial in this respect. Along these lines, it has been shown that the Vpr protein of SIVagm.Ver is able to antagonize a broad range of SAMHD1 proteins from primates (Lim et al., 2012). It is tempting to speculate that this may also be the case for SIVagm.Sab Vpr. This would favor the recombination (rather than the duplication) hypothesis, but would need to be assessed experimentally.

The rate of evolution of *vpx* seems to be slower than that of *vpr*, as shown by shorter branch lengths in the *vpx* lineage compared to the *vpr* one (see the HIV-2/SIVsmm/SIVmac lineage in Figure 5) (Tristem et al., 1992). This could be explained by the fact that *vpx* is overlapping with other genes to a greater degree than *vpr*, inducing a stronger constraint. In HIV-1 HXB2 for instance, 28% of the *vpr* gene is overlapping with other reading frames (*vif* and *tat*), whereas in HIV-2 BEN, 50% of *vpx* is overlapping (with *vpr*). A difference in selective pressures could also explain this disparity in evolution rate.

SIVcpz, which gave rise to the four known groups of HIV-1 after cross-species transmissions, is the result of recombination events between ancestors of SIVs from red-capped mangabeys and *Cercopithecus* species such as greater spot-nosed, mustached and mona monkeys (Bailes et al., 2003; Sharp and Hahn, 2011). Each of these viruses has the ability to antagonize SAMHD1 from the species they target (SIVrcm with *vpx* and SIVgsn/SIVmus/SIVmon with *vpr*) (Lim et al., 2012). However, this function was lost in SIVcpz (Etienne et al., 2013). As *vpx* from SIVrcm was unable to antagonize chimpanzee SAMHD1 (Lim et al., 2012), the loss of *vpx* might have had minimal consequences for the transmission of SIV to chimpanzees. It was hypothesized that the loss of *vpx*—*vpr* remained intact—led to the reconstruction of *vif* by overprinting and the acquisition of full antagonism of APOBEC3 proteins from chimpanzee (Etienne et al., 2013). One might speculate that evolution of *vif* to fully counteract chimpanzee APOBEC3G was more important for SIVcpz than evolution of *vpx* as a SAMHD1 antagonist.

Evolutionary analysis of SAMHD1 revealed strong signatures of positive selection in the N- and C-terminal parts of the protein (Laguette et al., 2012; Lim et al., 2012), suggesting that both termini may contribute to the interaction with Vpx. It has been shown recently that the separation of the two clades of *vpx* (HIV-2/SIVsmm/SIVmac and SIVrcm/SIVmnd-2) correlates with the domain of SAMHD1 targeted by these Vpx proteins. Vpx proteins from the HIV-2/SIVsmm/SIVmac lineage recognize the C-terminal domain of SAMHD1 (Ahn et al., 2012; Fregoso et al., 2013), whereas Vpx proteins from the SIVrcm/SIVmnd-2 lineage interact with the N-terminal domain of SAMHD1 (Fregoso et al., 2013; Wei et al., 2014). The model presented to explain this involves the particular head-to-tail dimer conformation of SAMHD1 and a Vpx (or Vpr) protein binding to both N-terminal and C-terminal domains of SAMHD1 with a high affinity for one of these domains and a lower affinity for the other. When mutations occurring in SAMHD1 lead to a decreased affinity and escape from Vpx, mutants of Vpx leading to higher affinity on the other domain might be selected (Fregoso et al., 2013). This evolutionary arms-race likely led to species specificity (Fregoso et al., 2013; Wei et al., 2014) and emphasizes the importance of the conservation of Vpx (Vpr) mediated SAMHD1 antagonism for lentiviruses. In agreement with this notion, SAMHD1 antagonism is actively maintained in natural infections, as exemplified by SIVagm *vpr* adaptations to SAMHD1 polymorphisms found in the African green monkey population (Spragg and Emerman, 2013).

## HIV-1, a pandemic virus lacking the X-traordinary protein

Although essential for certain primate lentiviruses, it is somewhat surprising that the ability to antagonize SAMHD1, as well as any other functions borne by *vpx*, were lost during the genesis of SIVcpz (Etienne et al., 2013). They were dispensable for the establishment of SIVcpz in chimpanzees and its cross-species transmission to humans, which gave rise to HIV-1. Whereas the other potential functions of *vpx* might be provided by other genes in HIV-1/SIVcpz, it seems that HIV-1 does not need to antagonize SAMHD1 to replicate efficiently in humans. It is still not understood why SAMHD1 antagonism is crucial in some primate lentiviruses but dispensable in others. HIV-1 mainly targets cycling CD4+ T cells in which dNTP concentrations are high (Diamond et al., 2004), hence SAMHD1 antagonism may not be needed. Of note, HIV-1 reverse transcriptase (RT) has a relatively high binding affinity for dNTPs, as compared to MLV (Weiss et al., 2004), which facilitates replication in cells with reduced dNTP content such as MDMs (Diamond et al., 2004). In line with this, HIV-1 RT mutant V148I, which has reduced dNTP binding affinity (Diamond et al., 2003, 2004), decreases HIV-1's ability to infect MDMs (Diamond et al., 2004; Lahouassa et al., 2012). Whether the RTs of HIV-2/SIVsmm have lower dNTP binding affinities than HIV-1 RT is currently unknown, but this could contribute to their dependence upon *vpx*. Alternatively viruses coding *vpx* might rely more on myeloid cell infection to disseminate *in vivo* than the ones lacking this gene. Of note, most non-primate lentiviruses, such as maedi-visna or caprine arthritis-encephalitis virus, tend toward myeloid tropism (though the mechanisms used by these viruses to cope with low dNTP levels are currently unknown).

It would be of major interest to determine whether there is a connection between SAMHD1 degradation and an altered equilibrium between pathogen replication and host survival. Indeed primate lentiviruses able to antagonize SAMHD1 seem better tolerated by their natural hosts. This is exemplified by the difference observed in pathogenicity between HIV-1 and SIVmnd-1 compared to HIV-2, SIVsmm and SIVmnd-2.

The *in vitro* infection of MDDCs with HIV-2 causes their activation through detection of viral cDNA by the DNA sensor cyclic GMP-APM synthase (cGAS) (Lahaye et al., 2013). In contrast, HIV-1 is normally unable to infect MDDCs and therefore does not activate them (Manel et al., 2010). However, HIV-1 can be artificially rendered able to infect DCs through the provision of Vpx, leading to potent MDDC activation and secretion of cytokines including type 1 IFN (Manel et al., 2010; Lahaye et al., 2013). Under these artificial conditions HIV-1 seems to cause a stronger MDDC activation than HIV-2 (Yu et al., 2013). This strongly suggests that the absence of Vpx may actually be beneficial for HIV-1 as this would help avoiding infection and activation of DCs and therefore prevent initial innate (and then adaptive) immune responses detrimental to the virus. This would not be a unique example of a virus benefiting from limiting myeloid cell tropism. Indeed mosquito-borne North American eastern equine encephalitis virus (EEEV) is potently restricted by a specific miRNA in myeloid cells, whereas the sequence targeted by this miRNA is crucial for mosquito vector infection (Trobaugh et al., 2013). By limiting myeloid cell tropism and consequent innate immunity induction, the restriction directly promotes neurologic disease manifestations characteristic of EEEV infection in humans. In line with this, it is tempting to speculate that HIV-2 might be less pathogenic because it induces innate immune responses, possibly through myeloid cell infection. However, *vpx* isolated from viremic and long-term aviremic HIV-2 infected individuals display similar abilities to antagonize SAMHD1 and enhance virus infection (Yu et al., 2013), presumably reflecting the complexity of viral pathogenesis.

Interestingly, there is some evidence that individuals presenting HIV-1/HIV-2 co-infections have better long-term outcomes and slower progression to AIDS, compared to HIV-1 mono-infected patients (Esbjornsson et al., 2012). HIV-2 co-infection seems to protect from HIV-1 pathogenesis for a certain period of time. It would be of high interest to understand the basis for this and whether Vpx plays a role in it.

## Conclusion

Vpx is a protein uniquely encoded by the HIV-2/SIVsmm and SIVrcm/SIVmnd-2 lineages, which has the property to favor myeloid cell infection through inducing the degradation of SAMHD1. It is absolutely required for efficient HIV-2/SIVsmm viral replication *in vivo*, suggesting an important role of myeloid cells as target cells for these viruses and/or that additional roles, such as APOBEC3A antagonism, may be important. In addition, Vpx is able to render DCs more permissive to HIV-1 infection, and this may promote the induction of stronger innate immune responses. These major findings might open the way for the prospective development of novel HIV-1 vaccine and treatment strategies based on the use of Vpx. In theory, treatment with a vaccine using Vpx-VLPs may stimulate immune responses, potentially leading to improved protection against (or control of) HIV-1.

### Conflict of interest statement

The authors declare that the research was conducted in the absence of any commercial or financial relationships that could be construed as a potential conflict of interest.
